# Capacity Choice in a Large Market

**DOI:** 10.1371/journal.pone.0101766

**Published:** 2014-08-18

**Authors:** Mats Godenhielm, Klaus Kultti

**Affiliations:** 1 Department of Economics, Hanken School of Economics, Helsinki, Finland; 2 HECER, Helsinki, Finland; 3 Department of Political and Economic studies, University of Helsinki, Helsinki, Finland; Universita' del Piemonte Orientale, Italy

## Abstract

We analyze endogenous capacity formation in a large frictional market with perfectly divisible goods. Each seller posts a price and decides on a capacity. The buyers base their decision on which seller to visit on both characteristics. In this setting we determine the conditions for the existence and uniqueness of a symmetric equilibrium. When capacity is unobservable there exists a continuum of equilibria. We show that the “best” of these equilibria leads to the same seller capacities and the same number of trades as the symmetric equilibrium under observable capacity.

## Introduction

We analyze endogenous capacity formation in a large market with frictions when the good for sale is perfectly divisible. The sellers post prices and decide on costly capacities. Buyers individually decide which seller to visit based on what is posted. This leads to the usual coordination frictions as the buyers don’t know which sellers the other buyers visit. This approach is called directed search. Standard references include [1]–[5].

We determine the conditions that guarantee the existence and uniqueness of a symmetric equilibrium both under free entry and when the measure of sellers is fixed. When capacities are observable, both price posting and auctions give rise to the same equilibrium quantities. When only prices are observable before the matching takes place, there is a continuum of equilibria. We show that the “best” of these equilibria yields the same seller capacity as the case of observable capacities and leads to the same number of traded goods. All equilibria under unobservable capacities give the sellers positive expected profits. Free entry of sellers therefore leads to a very large number of sellers each offering very small quantities. This is clearly inefficient and different than under observable capacity.

Two assumptions let us simplify the analysis compared to earlier papers. First, we focus on perfectly divisible goods. This is in contrast to several recent papers analyzing frictional markets and seller capacity for goods that are sold in units. (For example [5]–[9].) In these models, equilibrium is cumbersome to find when sellers can chose between more than two capacities. In [9], chap. 2 the existence of a free entry equilibrium under strongly convex costs is established. The equilibrium is straightforward to find, but uniqueness is not guaranteed. Second, we make the sellers' problem quasiconcave in a particular way by assuming convex costs but linear utility functions over capacity for the buyers. The linearity of the utility functions makes the distinction between capacity and quality somewhat arbitrary as any buyer who is willing to buy any amount given the unit price would buy everything a seller has to offer. As quality is seldom measured or priced in units we choose to frame the analysis in terms of quantity. Regardless, the setting resembles [10], with the distinction that we allow the sellers to chose any positive capacity/quality on the real line, whereas they study the choice between two levels.

## The Model

The environment consists of a unit interval of buyers and a large continuum of potential sellers of which 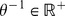 are active in the market. The overall market tightness, i.e., the ratio of buyers to active sellers, is 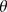. The sellers choose their capacity and post binding prices. The good is assumed to be perfectly divisible. Both the capacity and the price of each seller are observable. The cost of capacity 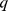 is 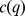, where 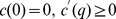 and 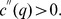 This cost is borne before the matching takes place as in [9], chap. 2 and [8].

The utility function of the buyers is linear, 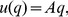
(1)where 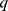 is the quantity that the buyer consumes and 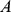 is a constant. The sellers choose their capacity and price so as to maximize their profit. Because the buyers' utility is linear and there is no upper bound on how much of a good a single buyer wants, a seller trades his whole quantity even if he is visited by just one buyer. Similarly, if two buyers contact a seller with capacity 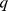 then they are indifferent between whether the good is divided equally between them or whether both get the whole quantity 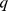 with probability 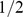. Thus the expected utility of a buyer visiting a seller with capacity 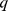 and unit price 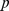 is simply 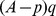 multiplied by the probability that the buyer ends up with quantity 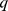 of the good.

The order of events is as follows: At stage 1, each active seller chooses a capacity 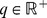 and bears the cost 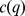. At stage 2, each seller posts a binding unit price 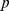, which depends on the distribution of capacities and his own capacity. At stage 3, each buyer chooses which seller to visit. There is perfect information as the actions of the previous stages are perfectly observed by the players. The symmetric equilibrium outcome would remain unchanged if the sellers were allowed to set both capacities and prices in the same stage, although new equilibria might arise. The real world motivation for the three stages is that revising pricing decisions is often easy whereas changing capacity/production is not. Consequently, sellers could always revise their prices after observing the capacities.

We capture the frictions by focusing on symmetric equilibrium strategies for the buyers. We further assume that the strategies of both the sellers and the buyers are anonymous so that sellers with the same capacity and the same price are treated identically by the buyers and all the buyers are treated identically by the sellers.

When there are different capacity-price pairs the buyers adjust their behavior so that they are indifferent between visiting the different types of sellers and expect the market utility 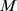 from them all. The idea can be traced back to [11],[3] and [4] and is often called the market utility approach. This adjustment of behavior leads to different ratios of buyers to sellers, i.e., queue lengths, 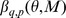, for sellers with different 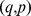 -pairs. When the queue length is 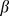 the probability that exactly 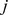 buyers visit a seller is given by the Poisson distribution, i.e., 
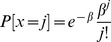
. Next we define the equilibrium. It consists of the following parts.

- At stage 1, all sellers choose their capacity 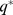 so as to maximize their profit given that they have to offer the buyers at least the market utility 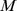


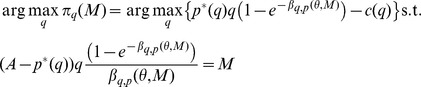

- At stage 2, all sellers choose their price 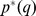 given their capacity 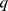, so that it maximizes their profit while giving the buyers the market utility 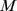


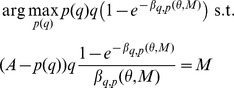

- At stage 3, buyers maximize their expected utility. Given a distribution of different capacity-price pairs 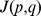 they adjust their behavior so that they are indifferent between visiting sellers with different 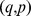 -pairs and expect to receive the same utility from all. This leads to different expected queue lengths 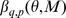 to the different types of sellers so that the expected utility of each buyer is 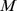 and 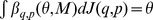.

**Definition 1** *Let* 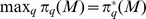. *A symmetric equilibrium is a capacity-price pair* 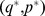, *a market utility* 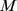, *and queue lengths* 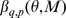 *such that* (i)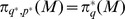; *(ii)* 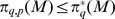 *for any other capacity-price pairs. (iii)* 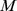 *and* 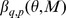 *constitute an equilibrium of the last stage subgame where each buyer contacts a seller, which means that the queue lengths* 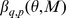 *are given by the market utility condition (part a)) whenever* 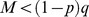, *and are set at zero whenever* 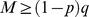. *In a symmetric equilibrium* 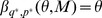 *and* 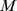 *is determined by* 
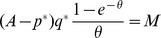
.

## Analysis

### Price formation

We begin by analyzing the second stage of the game where sellers have chosen their capacity. To find a symmetric equilibrium price, we first make the assumption that all sellers have the same capacity 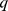 (we later show that this is the equilibrium outcome). The equilibrium price, if it exists, is then a unit price 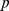 from which no seller has a profitable deviation. A possible deviator has the maximization problem 
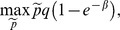
where 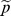 is his unit price. He sells his whole quantity 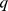 if he is visited by at least one buyer; the probability of which is 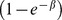. The queue length 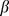 that the deviator faces is determined by the buyers' indifference condition between contacting the deviator and the non-deviators. This is the market utility condition: to get any buyers the deviator must offer at least the same expected utility as the non-deviators.
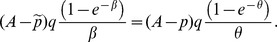



The LHS is the expected utility of a buyer visiting a deviating seller. As described in the set up, the linearity of the utility function allows us to write the expression for the expected utility as 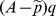 multiplied by the probability that the buyer ends up with the whole quantity 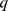 of the good. With probability 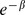 no other buyers show up and our buyer acquires quantity 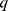. If 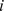 other buyers show up our buyer acquires 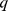 with probability 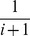. The probability that at least one other buyer shows up is 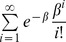. Thus the probability that our buyer manages to acquire 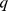 is 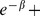 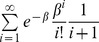, which simplifies to 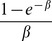. The RHS is the market utility or the expected utility of a buyer visiting the non-deviating firms. It is derived similarly as the LHS.

The first order condition of the deviating seller's problem is 
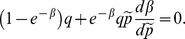



To find out how the queue length is affected by the price we totally differentiate the indifference condition of the buyers with respect to 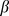 and 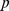 to get 
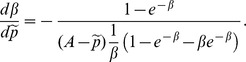



In equilibrium 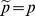 and 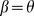, thus the first order condition implies that 
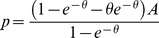
(2)


We show in the appendix that there are no profitable deviations from (2). Thus it is the equilibrium price.

**Proposition 2** *When all sellers have capacity* 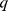 *the symmetric equilibrium price is given by* 
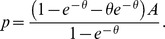


**Proof.** The first order conditions can be found above. The rest of the analysis is in the appendix S1. ▪

The equilibrium price above in (2) depends only on the overall market tightness 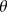. Capacity doesn’t enter the price because all sellers have the same capacity and the buyers have linear demands; therefore a seller sells his whole capacity even if visited by a single buyer. This does not mean that capacity is unimportant. To see why consider the case where there are sellers of two different capacities. For simplicity assume that proportion 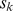 of sellers have capacity 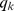 and the rest have capacity 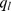. Then, following the steps above (for details consult the appendix S1.2), the equilibrium prices are 
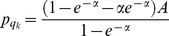
 and 
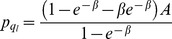
. Note that the difference in the price comes from the different queue lengths 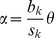, and 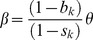, where 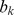 is the proportion of buyers visiting sellers with capacity 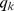. This proportion (and hence the queue lengths) is determined by the buyers' indifference condition 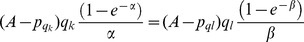 which depends on the sellers capacities.

#### Auctions

When all the sellers have capacity 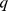 and post price 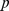 as per Proposition 2, their expected profit is 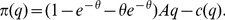
(3)


The buyers' expected utility is then 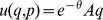
(4)


When the terms of trade are decided by auction at the sellers' locations a single buyer would bid zero and still acquire quantity 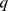 of the good. If there were two or more buyers they would compete for the good and thus bid up the per unit price to their valuation 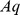. It is then easy to see that a buyer's expected utility from visiting a seller with quantity 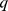 is 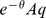 where 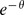 is the probability that no other buyers show up. By similar reasoning a seller receives a positive profit only if at least two buyers visit his auction. The probability that this happens is 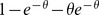. Thus the expected profit of an auction is given by 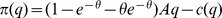. This is in essence the equivalence result from [12].

**Observation** The expected profits of the sellers are the same under posted prices and when trades are consummated by auction.

It turns out that the equivalence result also holds when there are sellers with differing capacities, just as in [9], chap. 2. The proof for the current setting with perfectly divisible capacities is in the appendix S1. As the auctions approach is easy to work with, we will use it to derive the equilibrium capacities of the sellers.

### Choice of capacity

With perfectly divisible goods it is straightforward to determine the conditions for existence and uniqueness of the symmetric equilibrium and to find the equilibrium. This is the main advantage compared to the approach with integer capacities. We proceed as in the last subsection. Namely, we assume that all sellers have capacity 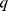 and analyze a potential deviator's problem and derive the queue length he faces by choosing capacity 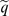. He must still offer the buyers the same expected utility, namely the market utility, that they would get from going to the sellers with capacity 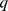. Thus the queue length of the deviator, i.e., 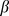 is determined by the buyers' indifference condition 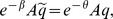



keeping in mind that the queue length cannot be negative. Simple algebra allows us to write the queue length 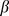, that a deviator faces as 
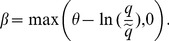
(5)


A seller deviating to capacity 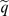 maximizes his expected profit 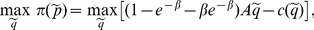
(6)where 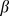 is determined by (5). The first order condition is 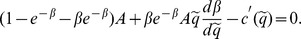



In order to solve the FOC we first derive
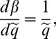
(7)which tells us how the expected queue length reacts to changes in capacity. In a symmetric equilibrium 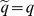 and 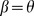. By substituting (7) into the first order condition we solve for 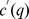. We get 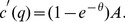
(8)


The necessary condition for equilibrium thus gives us 
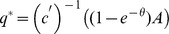



Unfortunately, it turns out that we need to make an extra assumption on the cost function in order to guarantee that the sufficient conditions hold, i.e., to show existence and uniqueness of the symmetric equilibrium. The reason is that the MR function of a potential deviator is increasing and concave in capacity. Therefore convexity of the cost function is not enough to guarantee that there are no profitable deviations. (The MC curve might be increasing and convex as well and might therefore cross the MR curve any number of times.) One way to guarantee existence and uniqueness is to assume that the MC increases more steeply than the MR curve after the potential equilibrium, but this is rather ad hoc as the condition then depends on the parameter values. A better way is to assume that 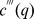 is non-negative. Then the MC curve is convex and the MR and MC curves cross at most twice and we can show that the sufficient conditions hold.

**Assumption A** 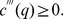


**Proposition 3** *The unique symmetric pure strategy equilibrium capacity of the sellers is given by 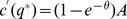 whenever this gives the sellers' a positive expected profit and assumption A holds*.

**Proof.** The necessary conditions are above, the sufficient conditions can be found in the appendix S1. ▪

It is somewhat surprising that Assumption A is needed for existence and uniqueness. The reason is that a seller can increase his queue length, and therefore the probability of trading, by deviating to a higher capacity. This in turn implies that the deviator has an increasing and convex revenue function. If the cost function is not convex enough, there might exist a profitable deviation to a high enough capacity. With a linear cost function no equilibrium exists as there is always a profitable deviation to a higher capacity just as in [9], chap. 2.

With Assumption A the equilibrium is straightforward to derive and easy to analyze. We elaborate with the following example.

**Example** When 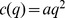 the equilibrium capacity is 
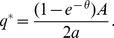
(9)


The sellers' profit from capacity 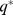 is 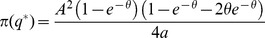
(10)


One notices that 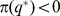 whenever 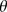 is smaller than or equal to the threshold 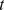 where 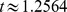 (as 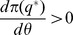 for 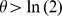 and 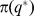 is continuous in 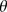 it follows that 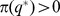 when 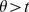). For 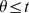 capacity 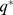 clearly isn’t an equilibrium as there exists a profitable deviation to 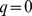. We can thus conclude that there exists no symmetric equilibrium in pure strategies when 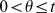. Whenever 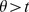 the equilibrium capacity of the sellers is given by proposition 3.

Even though no equilibrium with symmetric quantities for the sellers exist when 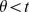, there exists an asymmetric equilibrium. In this equilibrium measure 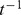 of the sellers have capacity 
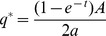
(11)and make zero profits and the rest of the sellers have capacity zero (or become inactive). For 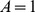 
and 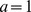 the equilibrium capacity for the active sellers is then 
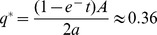


#### Constrained Efficiency

We analyze the efficiency of the decentralized equilibrium in a standard way by comparing it to the choice of a benevolent planner that maximizes overall utility. The planner chooses the capacities and the proportion of sellers offering each capacity and allocates the buyers over the sellers (see e.g. [13]). For the comparison to be fair it is assumed that the planner is constrained by the same frictions as the market participants. Namely, she cannot overcome the coordination problem by assigning specific buyers to specific sellers. If the planner cannot improve upon the market outcome the equilibrium is called constrained efficient. In this subsection we assume that the overall measure of sellers is fixed and not a choice variable of the planner.

We define social welfare directly as the expected value of the trades minus the sellers' capacity cost. We show in the appendix that the planner has no solution in which there are sellers with different (positive) capacities. When all sellers have the same capacity the social welfare is 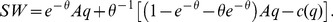
(12)


The planner maximizes welfare by choosing the capacity of the sellers. As noted above, she can’t base her decisions on the identities of the agents. Assuming that she chooses the same capacity for all sellers, the planner's problem is 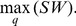
(13)


The FOC is 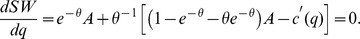



Solving for 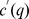 we get 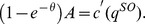



To see that 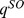 uniquely maximizes social welfare note that 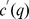 is non decreasing, 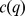 is continuous and 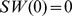 and 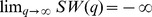. In addition 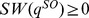 for any non negative value of 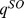. The competitive outcome is thus identical to the planners solution or 
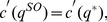
(14)whenever the overall market tightness is such that the competitive solution gives the sellers a non negative expected profit or 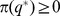.

### Free entry

Equilibrium is constrained efficient when the number of sellers is determined by free entry. In this case the measure of sellers, 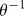, adjusts so that their zero profit condition is satisfied. Thus for any 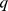


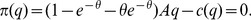
(15)


From Eq. (8) we know that in any equilibrium 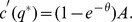



With the two equations we can solve for the free entry equilibrium. Returning to our example with cost function 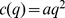 the free entry equilibrium capacity is given by (11). The measure of sellers is the solution in 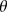 to
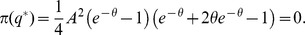



We find that 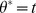, where 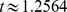 just as earlier in the case with too many sellers. Thus, in a free entry equilibrium measure 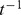 of the sellers have capacity 
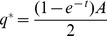
 and the rest have capacity zero.

#### Planner's solution

When the social planner is free to chose both the overall market tightness and the capacity of the sellers the social optimum is given by 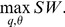
(16)


The FOC's are 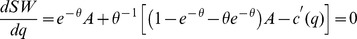
and
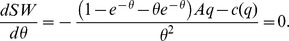



Rearranging we get 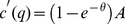
(17)and 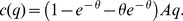
(18)


As (17) and (18) are identical to (8) and (15) the free entry equilibrium is constrained efficient.

**Proposition 4** *The symmetric equilibrium is constrained efficient*

**Proof.** The proof can be found above. ▪

Constrained efficiency is not a surprising result. It is almost a defining property of directed search and has been demonstrated several times with fixed capacities and free entry. Here capacity is not fixed but it is observable and there is an optimal price for each capacity. Thus capacity choices are reflected in the queue lengths and hence in the trading probabilities of the sellers. With free entry the sellers fully internalize the effect their decisions have on welfare. Analogous results can be found for example in [9], chap. 2, [8] and [7].

Our results on the constrained efficiency of equilibrium are a useful benchmark in the following section where capacities are unobservable.

### Unobservable capacity

In this section we let the sellers' capacities be unobservable before matching takes place. The definition of equilibrium from section 2.1 needs to be changed accordingly. To derive the queue lengths we need to describe the beliefs. The standard way is to impose strict beliefs of the type that all sellers that post price 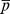 have a “high” capacity and others have “low” capacity. With continuous capacities this can be modified to: all sellers that post price 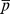 have capacities that maximize their profit and therefore satisfy 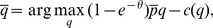
(19)i.e., where 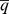 is determined by 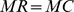 or 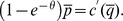 Any seller posting any other price than 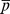 is assumed to have capacity zero. In the candidate equilibrium the price is therefore 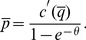
(20)


The equilibrium capacity 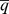 is 
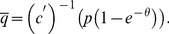
(21)


Unfortunately there is a continuum of equilibria satisfying these beliefs as any 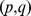 pair such that 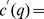 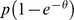 and 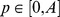 yields non negative expected payoffs and can be supported as equilibrium. Refinements such as the Cho-Kreps intuitive criterion have no bite.

#### Equilibrium selection

Let us first focus on the equilibrium capacity that maximizes social welfare. We define social welfare as the overall utility from trade minus the costs of production or 
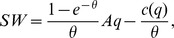
where the first term on the RHS is a buyer's expected utility multiplied with the measure of buyers. The second term is the cost of capacity 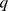 multiplied by 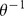, i.e., the measure of sellers. We find the “best” equilibrium by maximizing 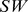 with respect to 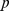. By substituting (20) and (21) in the social welfare function the maximization can be written as 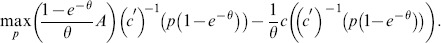
(22)


The FOC is
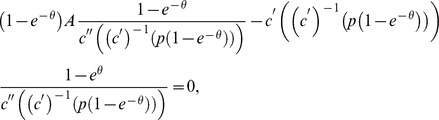
which can be simplified to
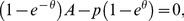
(23)which holds when
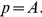
(24)


The equilibrium capacity is then given by 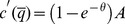
(25)just as when capacities are observable. The price 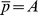 is, however, much higher than than under observable capacities. In fact all the gains from trade befall the sellers while the buyers get zero utility.

#### Auctions

When trade is determined by auction (without reserve price) the equilibrium capacity is simply
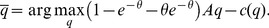
(26)which gives us 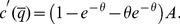
(27)


The unique symmetric equilibrium capacity under auctions is given by (27). It is lower than (25), the capacity in the “best” equilibrium. To further analyze the differences between the different cases we again let the cost function be given by 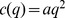.

**Example (continued)**: The equilibrium capacity under auctions is smaller than in the “best” equilibrium under price posting. 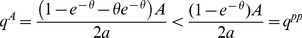
(28)


This is not surprising as the gains from trade are divided more equally between the market participants under auctions but the capacity costs are still borne by the sellers. It is likewise clear that the expected profits of the sellers are higher in the “best” price posting equilibrium 
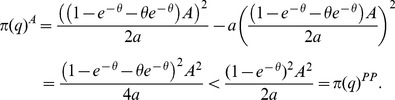
(29)


The buyers' expected utility is, on the other hand, zero in the “best” price posting equilibrium whereas it is positive under auctions. 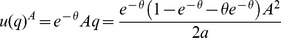
(30)


The “best” equilibrium under price posting and unobservable capacity achieves the same welfare as the symmetric equilibrium under observable capacities. In doing so it allocates all the gains from trade to the sellers. Auctions result in lower equilibrium capacities than the “best” price posting equilibrium, but the benefits of trade are more evenly distributed by the market participants.

By substituting the equilibrium capacity under auctions 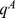 (unobservable capacities) into (20), and imposing suitable beliefs, we solve for the price that yields the same equilibrium capacity as auctions. This price is 
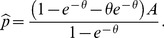
(31)


Interestingly 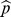 is identical to the symmetric equilibrium unit price under observable capacity (see Proposition 2). Just as under observable capacity, it results in both the buyers and the sellers receiving the same expected utilities as under auctions.

Above we analyze capacity choice when only prices are observable. To do so we impose strict beliefs. The downside of this assumption is that it kills any interesting link between queue length and capacity. In addition, it gives rise to a continuum of equilibria of which we focus on two. The “best” equilibrium maximizes social welfare and leads to the same capacities as under observable capacity. The reason is that now the whole surplus of trade befalls the sellers and hence they fully internalize the effect of their capacity decisions. The equilibrium is therefore constrained efficient with a fixed number of sellers.

In the second equilibrium the trades are determined by auction. The equilibrium leads to too small capacities compared to observable capacity. Somewhat interestingly it is outcome-wise equivalent to one where sellers post the same price as under observable capacity.

## Conclusion

Directed search is a standard method to analyze frictional markets. At its core is the trade-off that sellers face between asking a higher price and attracting fewer buyers; hence trading more slowly. Typically, all sellers are assumed to have a fixed capacity, often one unit. Several recent papers relax this assumption by allowing the sellers to choose their capacity. This makes it possible to compare markets with a few large sellers to markets with many small sellers in terms of welfare and to find the equilibrium size and number of sellers given the cost function. In the realistic setting where production takes place before trading these models usually yield equilibria that can be analyzed only numerically (see [9], chap. 2). In the current paper we simplify the setting by letting the goods be perfectly divisible and the buyers' utility functions be linear. For a unique symmetric equilibrium to exist we still have to assume a very convex cost function. The gain is that the equilibrium is straightforward to analyze and easy to work with whether capacities are observable or not.

Were we to relax the assumption of linear demands, for example, by assuming that buyers have diminishing marginal utilities even the observable capacities case would be quite cumbersome to analyze as can be seen e.g. in [9], chap. 3. The analysis is, while interesting, outside the scope of the current paper and is left for future work.

## Supporting Information

Figure S1 **The MR curve and the MC curve of a single seller.**
(TIF)

Appendix S1 **Omitted proofs.**
(PDF)
