# Supplementary figures and images for: Capacity Choice in a Large Market
Source: PLoS One. 2014 Aug 18;9(8):e101766. doi: 10.1371/journal.pone.0101766 (PMC4136719; doi:10.1371/journal.pone.0101766)

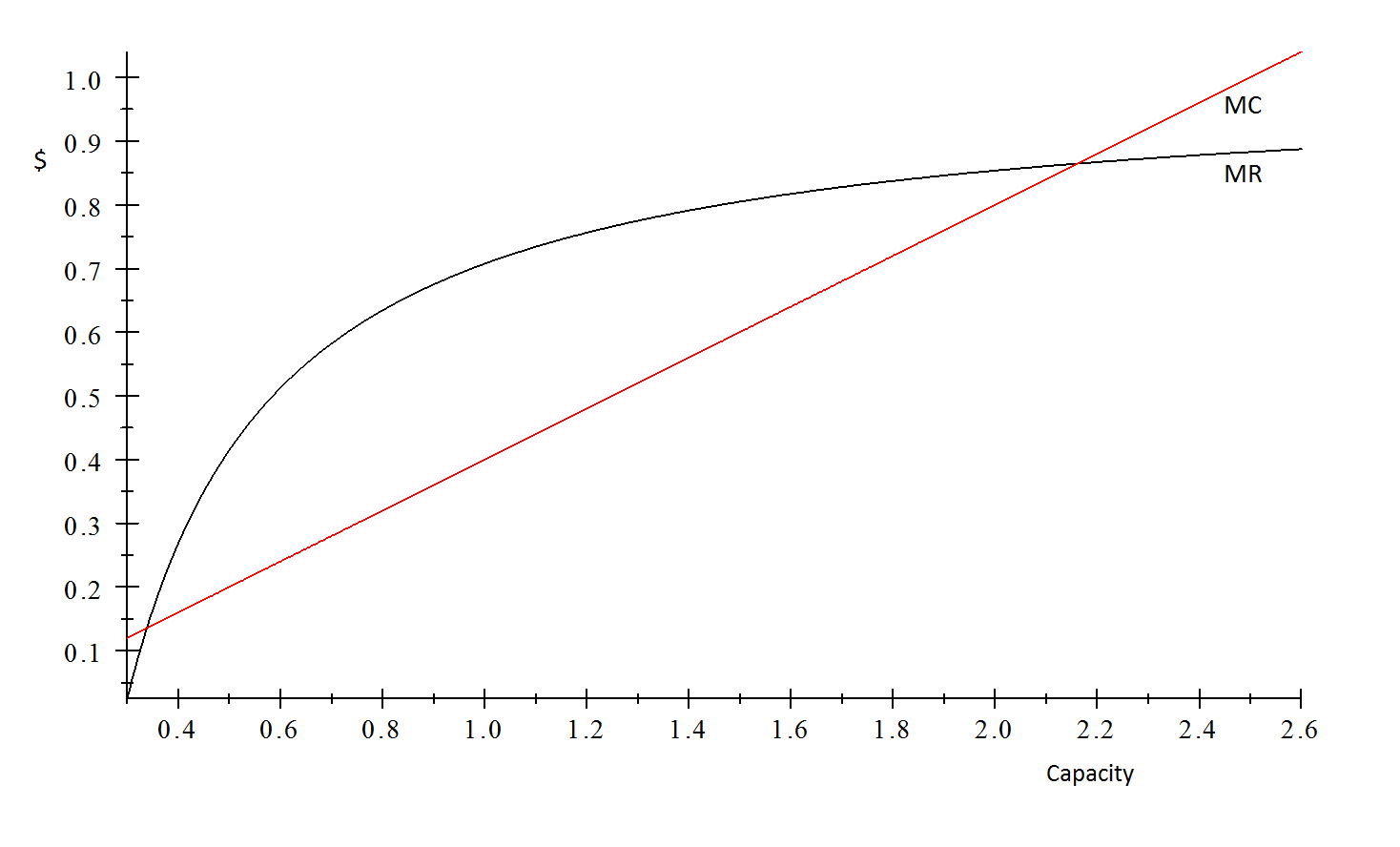

Supplement: Figure S1 — The MR curve and the MC curve of a single seller. (TIF) [file pone.0101766.s001.tif]
