# Supplementary material for: Capacity Choice in a Large Market
Source: PLoS One. 2014 Aug 18;9(8):e101766. doi: 10.1371/journal.pone.0101766 (PMC4136719; doi:10.1371/journal.pone.0101766)
Supplement: Appendix S1 — Omitted proofs. (PDF) [file pone.0101766.s002.pdf]

Appendix S1: Omitted Proofs and Results

S1.1 Sufficient conditions for Proposition 2

Proof. When the price is $p = \frac{\left(1-e^{-\theta}-\theta e^{-\theta}\right)A}{1-e^{-\theta}}$ the expected revenue of a seller with capacity q is given by

$$pq\left(1-e^{-\theta}\right) = (1-e^{-\theta}-\theta e^{-\theta})Aq.$$

Deviating to some other price $\widetilde{p}$ gives

$$\widetilde{p}q\left(1-e^{-\beta}\right),$$

where the queue length of a deviator is determined by the buyers indifference condition

$$(A-p)\frac{q}{\theta}\left(1-e^{-\theta}\right) = (A-\widetilde{p})\frac{q}{\beta}\left(1-e^{-\beta}\right).$$

Rewriting we get

$$e^{-\theta}Aq = (A-\widetilde{p})\frac{q}{\beta}\left(1-e^{-\beta}\right).$$

Thus

$$\widetilde{p} = \frac{A\left(\frac{q}{\beta}\left(1-e^{-\beta}\right)-qe^{-\theta}\right)}{\frac{q}{\beta}(1-e^{-\beta})}.$$

A deviator's expected revenue is

$$\widetilde{p}q\left(1-e^{-\beta}\right) = \frac{A\left(\frac{q}{\beta}\left(1-e^{-\beta}\right)-qe^{-\theta}\right)}{\frac{q}{\beta}(1-e^{-\beta})}q\left(1-e^{-\beta}\right) = Aq\left(1-e^{-\beta}-\beta e^{-\theta}\right).$$

For p to be the equilibrium price it must give a potential deviator a higher profit than any other price would give him. Thus the following inequality must hold

$$(1-e^{-\theta}-\theta e^{-\theta}) \geq (1-e^{-\beta}-\beta e^{-\theta}).$$

To find the maximum of the RHS we first differentiate it with respect to β.

$$\frac{d}{d\beta}(1-e^{-\beta}-\beta e^{-\theta}) = e^{-\beta}-e^{-\theta}$$

This is zero when $\beta = \theta$. The second order condition with respect to β is negative. As the RHS is continuos in β and the first derivative is zero only when $\beta = \theta$ it follows that $\beta = \theta$ gives the global maximum. Thus there is no profitable deviation from price $p = \frac{\left(1-e^{-\theta}-\theta e^{-\theta}\right)A}{1-e^{-\theta}}$. ■

S1.2 Payoff equivalence of posted priced and auctions with heterogenous sellers

When there are sellers of several different capacities q_i the equilibrium prices are derived the same way as in subsection 2.1. The buyers have to be indifferent between all the different sized sellers and expect the market utility M from any seller. We consider a seller with capacity q_j who considers deviating from price p_j to some price $\widetilde{p}_j$. We denote the queue length that the deviator faces by β_j and the queue length that the nondeviators face by θ_j. A buyer visiting the deviator also expects to receive the market utility M.

$$(A - \widetilde{p}_j) q_j \frac{\left(1 - e^{-\beta_j}\right)}{\beta_j} = M$$

By repeating the steps of the identical capacities case get the first order condition of a deviator with capacity q_j as

$$\left(1 - e^{-\beta_j}\right) q_i + e^{-\beta_j} q_j \widetilde{p}_j \frac{d\beta_j}{d\widetilde{p}_j} = 0.$$

To find out how the queue length is affected by the price we totally differentiate the indifference condition of the buyers with respect to β_j and $\widetilde{p}_j$ to get

$$\frac{d\beta_j}{d\widetilde{p}_j} = -\frac{1 - e^{-\beta_j}}{(A - \widetilde{p}_j)\frac{1}{\beta_j}\left(1 - e^{-\beta_j} - \beta_j e^{-\beta_j}\right)}.$$

In equilibrium $\widetilde{p}_j = p_j$ and $\beta_j = \theta_j$, thus the first order condition implies that

$$p_j = \frac{\left(1 - e^{-\theta_j} - \theta_j e^{-\theta_j}\right) A}{1 - e^{-\theta_j}}$$

The sufficient conditions are almost identical to those for Proposition 1 and are hence omitted. The queue lengths θ_i capture the ratio of buyers to sellers with a given size q_i. They are determined by the market utility condition. In addition, the overall measure of buyers sums up to unity and the overall measure of sellers sums up to θ^{-1} so that the overall market tightness remains θ.

With the equilibrium prices determined as above it is easy to see that auctions and posted prices are payoff equivalent to all agents also when there are sellers of different sizes. Assuming e.g. two different capacities q_k and q_l the indifference condition for the buyers under both trading mechanisms is simply

$$e^{-\theta_k} A q_k = e^{-\theta_l} A q_l.$$

The expected revenue of sellers with capacity q_k is under both trading mechanisms $\left(1 - e^{-\theta_k} - \theta_k e^{-\theta_k}\right) A q_k$ and the expected revenue of sellers with capacity q_l is $\left(1 - e^{-\theta_l} - \theta_l e^{-\theta_l}\right) A q_l$. In both cases the buyers distribute themselves so that the indifference condition between their expected utilities from visiting the different types of sellers is satisfied, this behavior determines θ_k and θ_l uniquely.

S1.3 Sufficient conditions for Proposition 3

Proof. Let (8) hold and the corresponding expected profit be non-negative. The prospective symmetric equilibrium is then given by $c'(q) = \left(1 - e^{-\theta}\right) A$. A seller deviating to $\widetilde{q}$ while offering the same market utility can expect a queue length determined by $\beta = \max\left(\theta - \ln(\frac{q^*}{\widetilde{q}}), 0\right)$ as per (5). The expected profit of the deviator is

$$(1 - e^{-\beta} - \beta e^{-\beta}) A \widetilde{q} - c(\widetilde{q}) \tag{S-1}$$

Differentiating the profit with respect to $\widetilde{q}$ we get

$$\beta e^{-\beta} \frac{1}{\widetilde{q}} A \widetilde{q} - (1 - e^{-\beta} - \beta e^{-\beta}) A - c'(\widetilde{q}) =$$

$$\left(\theta - \ln(\frac{q^*}{\widetilde{q}})\right) e^{-\theta} \frac{q^*}{\widetilde{q}} A + \left(1 - e^{-\theta} \frac{q^*}{\widetilde{q}} - \theta e^{-\theta} \frac{q^*}{\widetilde{q}} + \ln\left(\frac{q^*}{\widetilde{q}}\right) e^{-\theta} \frac{q^*}{\widetilde{q}}\right) A - c'\left(\widetilde{q}\right),$$

which further simplifies to

$$\left(1 - e^{-\theta} \frac{q^*}{\widetilde{q}}\right) A - c'(\widetilde{q}) \tag{S-2}$$

When $\widetilde{q} = q^*$ the first order condition (S-2) is zero. This is the prospective symmetric equilibrium. The second order condition is $\frac{e^{-\theta} A}{q^*} - c''(\widetilde{q})$, which might be negative or positive depending on the exact curvature of $c(\widetilde{q})$. The MR curve of the deviator (the first term in (S-2)) is increasing and concave. The MC curve (the second term in (S-2)) is also increasing and might cross the MR curve more than twice. Then there might exist large profitable deviations for the deviator even if the SOC holds. To guarantee

that the MR and MC curves cross at most two times we must impose further conditions on $c(\widetilde{q})$. If $c'''(\widetilde{q}) \geq 0$, then the MR curve is convex and crosses the MC curve either twice (when the MR curve is below the MC curve at the smallest value of $\widetilde{q}$) or once. Because the deviator must offer the buyers the market utility in order to attract any buyers the MR curve is always zero until the smallest value of $\widetilde{q}$ for which $\beta = \theta - \ln(\frac{q^*}{\widetilde{q}})$ is non-negative and hence the MR and MC curves cross twice for $\widetilde{q} = q^*$ to be the global maximum. Then q^* is the symmetric equilibrium capacity whenever leading to non-negative expected profit for the sellers. ■

Figure S1 below illustrates the MR curve of the deviator offering the buyers the market utility and the MC curve when $c(q) = aq^2$, $a = 0.2$, $A = 1$, and $\theta = 2$.

S1.4 Proof that there cannot be several capacities in the planner's solution

We show below that the planner doesn't have a solution where she puts weight on more than one capacity.

Proof. Assume that there are two capacities q_1 and q_2 in the planner's solution. The welfare function is then

$$\begin{aligned} SW &= be^{-\frac{b}{s}\theta}Aq_1 + s\theta^{-1}\left[\left(1 - e^{-\frac{b}{s}\theta} - \frac{b}{s}\theta e^{-\frac{b}{s}\theta}\right)Aq_1 - c(q_1)\right] + (1-b)\,e^{-\frac{(1-b)}{(1-s)}\theta}Aq_2 \\ &+ (1-s)\,\theta^{-1}\left(1 - e^{-\frac{(1-b)}{(1-s)}\theta} - \frac{(1-b)}{(1-s)}\theta e^{-\frac{(1-b)}{(1-s)}\theta}\right)Aq_2 - c(q_2)w \\ &= s\theta^{-1}\left(1 - e^{-\frac{b}{s}\theta}\right)Aq_1 - s\theta^{-1}c(q_1) + (1-s)\,\theta^{-1}\left(1 - e^{-\frac{(1-b)}{(1-s)}\theta}\right)Aq_2 - (1-s)\theta^{-1}c(q_2), \end{aligned} \tag{S-3}$$

where s is the proportion of sellers with capacity q_1 and b is the proportion of buyers visiting these sellers. The proportions are the solution to the planner's problem.

$$\max_{b,s}\ \left[\ s\theta^{-1}\left(1 - e^{-\frac{b}{s}\theta}\right)Aq_1 - s\theta^{-1}c(q_1) + (1-s)\,\theta^{-1}\left(1 - e^{-\frac{(1-b)}{(1-s)}\theta}\right)Aq_2 - (1-s)\theta^{-1}c(q_2)\right].$$

The first order condition w.r.t. b is

$$\theta^{-1}\left(e^{-\frac{b}{s}\theta}Aq_1 - e^{-\frac{(1-b)}{(1-s)}\theta}Aq_2\right) = 0,$$

which gives us

$$e^{-\frac{b}{s}\theta} q_1 = e^{-\frac{(1-b)}{(1-s)}\theta} q_2. \tag{S-4}$$

The first order condition w.r.t. s is

$$\theta^{-1}\left[\left(1 - e^{-\frac{b}{s}\theta} - \frac{b}{s}\theta e^{-\frac{b}{s}\theta}\right) Aq_1 - c(q_1) - \left(1 - e^{-\frac{(1-b)}{(1-s)}\theta} - \frac{(1-b)}{(1-s)}\theta e^{-\frac{(1-b)}{(1-s)}\theta}\right) Aq_2 + c(q_2)\right] = 0,$$

which gives us

$$\left(1 - e^{-\frac{b}{s}\theta} - \frac{b}{s}\theta e^{-\frac{b}{s}\theta}\right) Aq_1 - c(q_1) = \left(1 - e^{-\frac{(1-b)}{(1-s)}\theta} - \frac{(1-b)}{(1-s)}\theta e^{-\frac{(1-b)}{(1-s)}\theta}\right) Aq_2 - c(q_2). \tag{S-5}$$

Now let $\gamma = \frac{b}{s}\theta$ and $\overline{\gamma} = \frac{1-b}{1-s}\theta$ be fixed and assume that they solve (S-4) and (S-5). For (S-3) to be the welfare associated with the planner's solution the capacities q_1 and q_2 must maximize social welfare given the fixed proportions s and b. We note the planner's maximization problem can then be separated to

$$\begin{aligned} &\max_{q_1,q_2}\left[s\theta^{-1}\left(1-e^{-\gamma}\right)Aq_1 - s\theta^{-1}c(q_1) + (1-s)\,\theta^{-1}\left(1-e^{-\overline{\gamma}}\right)Aq_2 - (1-s)\theta^{-1}c(q_2)\right] \\ &= \max_{q_1}\left[s\theta^{-1}\left(1-e^{-\gamma}\right)Aq_1 - s\theta^{-1}c(q_1)\right] + \max_{q_2}\left[(1-s)\,\theta^{-1}\left(1-e^{-\overline{\gamma}}\right)Aq_2 - (1-s)\theta^{-1}c(q_2)\right]. \end{aligned} \tag{S-6}$$

We focus on the first part of the problem and solve

$$\max_{q_1}\left[s\theta^{-1}\left(1-e^{-\gamma}\right)Aq_1 - s\theta^{-1}c(q_1)\right]. \tag{S-7}$$

The FOC is

$$s\theta^{-1}\left(1-e^{-\gamma}\right)A - s\theta^{-1}c'(q_1) = 0$$

which gives

$$\left(1-e^{-\gamma}\right)A = c'(q_1^{SO}). \tag{S-8}$$

Note that (S-8) is equivalent to the equilibrium condition in Proposition 3. Note, in addition, that

the expressions (S-4) and (S-5) correspond to the buyers' respective sellers' indifference conditions in an equilibrium (of a decentralized economy) where there are sellers of capacities q_1 and q_2. But we have already shown, (in the proof of proposition 3), that the equilibrium is unique if the cost function satisfies assumption A. Thus there cannot exist a capacity q_2 such that (S-4) and (S-5) both hold. Therefore there cannot exist a planner's solution where there are two capacities. The generalization of the proof to $n > 2$ capacities is trivial and is therefore omitted. ■
